# Multifacility Outbreak of Middle East Respiratory Syndrome in Taif, Saudi Arabia

**DOI:** 10.3201/eid2201.151370

**Published:** 2016-01

**Authors:** Abdullah Assiri, Glen R. Abedi, Abdulaziz A. Bin Saeed, Mutwakil A. Abdalla, Malak al-Masry, Abdul Jamil Choudhry, Xiaoyan Lu, Dean D. Erdman, Kathleen Tatti, Alison M. Binder, Jessica Rudd, Jerome Tokars, Congrong Miao, Hussain Alarbash, Randa Nooh, Mark Pallansch, Susan I. Gerber, John T. Watson

**Affiliations:** Ministry of Health, Riyadh, Saudi Arabia (A. Assiri, A.A. Bin Saeed, M. al-Masry);; Centers for Disease Control and Prevention, Atlanta, Georgia, USA (G.R. Abedi, X. Lu, D.D. Erdman, K. Tatti, A.M. Binder, J. Rudd, J. Tokars, C. Miao, M. Pallansch, S.I. Gerber, J.T. Watson);; Taif Governorate Health Directorate, Taif, Saudi Arabia (M.A. Abdalla);; Field Epidemiology Training Program, Ministry of Health, Riyadh (A.J. Choudhry, H. Alarbash, R. Nooh)

**Keywords:** Middle East respiratory syndrome, coronavirus, epidemiology, genetic sequencing, serum, viruses, Saudi Arabia, MERS, MERS-CoV, respiratory infections

## Abstract

Middle East respiratory syndrome (MERS) coronavirus (MERS-CoV) is a novel respiratory pathogen first reported in 2012. During September 2014–January 2015, an outbreak of 38 cases of MERS was reported from 4 healthcare facilities in Taif, Saudi Arabia; 21 of the 38 case-patients died. Clinical and public health records showed that 13 patients were healthcare personnel (HCP). Fifteen patients, including 4 HCP, were associated with 1 dialysis unit. Three additional HCP in this dialysis unit had serologic evidence of MERS-CoV infection. Viral RNA was amplified from acute-phase serum specimens of 15 patients, and full spike gene-coding **sequencing **was obtained from 10 patients who formed a discrete cluster; sequences from specimens of 9 patients were closely related. Similar gene sequences among patients unlinked by time or location suggest unrecognized viral transmission. Circulation persisted in multiple healthcare settings over an extended period, underscoring the importance of strengthening MERS-CoV surveillance and infection-control practices.

## Abstract 2

Enhanced surveillance and infection-control practices are needed to prevent outbreaks in healthcare settings.

Middle East respiratory syndrome (MERS) coronavirus (MERS-CoV) is a novel betacoronavirus associated with a broad spectrum of respiratory illness; infection results in death in ≈35%–40% of cases (*1*). Since the virus was first identified 2012, more than 85% of cases have occurred in Saudi Arabia (*1*). Although risk factors for transmission have not been well described, camels (*Camelus dromedarius*) are suspected reservoirs, as suggested by case investigations (*2**,**3*), serologic studies (*4**,**5*), and isolation of live infectious MERS-CoV (*2**,**3**,**6*). Limited human-to-human transmission has been documented in households (*7*) and healthcare facilities (*8**–**10*), but no sustained community transmission has been documented (*1*). In Jeddah in 2014, secondary transmission (i.e., from infected to noninfected persons) accounted for 97% of assessed cases (*9*).

Although detection of MERS-CoV RNA from persons with mild symptoms, typically in healthcare personnel (HCP), is well-documented (*11*), the potential role that mild cases play in transmission is not well defined (*12*). In healthcare facilities, extensive transmission of MERS-CoV in dialysis units has been documented (*8**,**9*); in those events, strengthening infection-control precautions preceded decreased numbers of reported cases. Currently, the surveillance case definition for MERS in Saudi Arabia requires the presence of symptoms (*13*), and testing is reserved primarily for symptomatic patients, often with severe illness.

MERS cases were first reported from Taif Governorate (population 1.1 million) in the Makkah Region of Saudi Arabia in June 2013, and 15 cases were reported during June 2013–June 2014. Beginning in September 2014, additional cases of MERS were reported from multiple healthcare facilities in Taif, including a cluster associated with a dialysis unit. The Saudi Arabia Ministry of Health (MoH), assisted by the US Centers for Disease Control and Prevention (CDC), began an investigation to determine the cause and scope of the outbreak, epidemiologic links between patients, and epidemiologic and clinical features of patients.

## Methods

### Setting

Hospital A is a 368-bed tertiary acute-care facility and serves military staff and their families. Hospital B is a 500-bed tertiary MoH hospital with an associated but physically separate outpatient renal dialysis unit. Hospital C is a 250-bed MoH facility and is the MERS-CoV designated referral hospital for Taif. Hospital D is a private hospital.

### Epidemiologic Investigation

We defined a case-patient as any patient from Taif Governorate who was reported with laboratory-confirmed MERS-CoV infection during August 1, 2014–February 1, 2015. In Saudi Arabia, reporting is required for all patients with clinical or radiologic evidence compatible with MERS-CoV disease and with a positive real-time reverse transcription PCR (rRT-PCR) on 2 specific gene targets: the region upstream of the E gene and open reading frame 1a (*13*). We reviewed available medical and public health records for all reported case-patients during the study period and conducted interviews with available hospital staff. We collected available demographic information, medical history, symptoms at onset, clinical course, preillness exposures, and evaluation and treatment locations. We grouped together case-patients whose illness onset occurred within 2–14 days of exposure (work- or treatment-related) to the same facility. Available residual patient specimens were analyzed at CDC.

### Laboratory Investigation

#### Molecular Detection and Gene Sequencing

At the MoH Regional Laboratory at Makkah, rRT-PCR testing for MERS-CoV RNA was performed on nasopharyngeal (NP) specimens. Serum specimens collected from laboratory-confirmed case patients were sent to CDC for MERS-CoV serology and were tested for viremia by rRT-PCR (*14*). Positive respiratory specimens were not retained and thus unavailable for confirmatory rRT-PCR testing or sequencing at CDC.

Sequencing of the coding region of the spike protein gene (4,062 nt) was performed by using a 3130xl Genetic Analyzer (Applied Biosystems, Grand Island, NY, USA); analysis was performed by using Sequencher 4.8 (Gene Codes, Ann Arbor, MI, USA) for sequence assembly and editing. Sequence alignments were prepared by using ClustalX 1.83 (http://www.clustal.org/) and implemented in BioEdit 7.2.5 (http://www.mbio.ncsu.edu/BioEdit/page2.html). Phylogenetic analyses were performed by using MEGA 6.06 (http://www.megasoftware.net). The neighbor-joining method (tree algorithm inferred with the Kimura 2-parameter substitution model of sequence evolution) was used to construct phylogenetic trees, and bootstrap resampling analysis was performed (1,000 replicates) to test tree-branching significance.

#### Serologic Assessment

MERS-CoV antibody positivity was defined as a positive result from screenings of MERS-CoV nucleocapsid ELISA and confirmatory positive results by immunofluourescence and microneutralization assays, as described (*15*). A serosurvey of HCP who were exposed to confirmed MERS-CoV patients in the dialysis unit of hospital B was conducted 3 weeks after the period of suspected transmission (Technical Appendix).

### Statistical Analysis

For reported demographic and clinical characteristics, differences were assessed for significance (p = 0.05) by using χ^2 ^test, Fisher exact test, and *t*-test, as appropriate. All data were analyzed by using SAS 9.3 (SAS Institute, Cary, NC, USA).

## Results

### Epidemiologic Investigation

**Figure 1:**
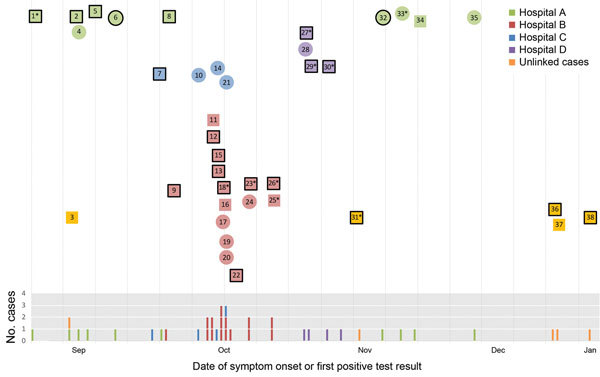
Middle East respiratory syndrome coronavirus (MERS-CoV) case-patients reported in Taif, Saudi Arabia, during September 2014–January 2015. Indicated are time of symptom onset or first positive laboratory testing. Healthcare setting where transmission likely occurred is shown by color. Circles indicate healthcare personnel (HCP), squares non-HCP; black outlines indicate that patient died. Asterisks (*) indicate that sequencing was performed on the patient’s serum sample.

During August 1, 2014–February 1, 2015, the MoH received reports of 38 patients with laboratory-confirmed MERS-CoV (Figure 1). Twenty-eight (74%) were men, 22 (58%) were of Saudi nationality, and median age was 51 (range 17–84) years (Table 1). Thirteen (34%) patients were HCP: 7 nurses, 2 physicians, 2 cleaning personnel, 1 administrative professional, and 1 clerk. The most common underlying medical conditions were diabetes, reported by 16 (47%), and renal failure requiring dialysis, reported by 12 (33%). At illness onset, 35 (92%) patients reported >1 respiratory symptom. Two patients, both HCP identified through routine testing of contacts of previously identified patients, reported no symptoms (Table 1).

**Table 1:** Demographic and clinical characteristics of patients with laboratory-confirmed Middle East respiratory syndrome coronavirus infection, Taif, Saudi Arabia, August 2014–February 2015*

Characteristic	Patients, n = 38	Survived, n = 17	Died, n = 21	p value
Male sex	28/38 (74)	9/17 (53)	19/21 (90)	0.023
Nationality				
Saudi	22/38 (58)	6/17 (35)	16/21 (76)	0.020
Non-Saudi	16/38 (42)	11/17 (65)	5/21 (24)	
Occupation				
Healthcare personnel	13/38 (34)	11/17 (65)	2/21 (10)	<0.001
Non–healthcare personnel	25/38 (66)	6/17 (35)	19/21 (90)	
Underlying medical conditions or behaviors				
Renal failure requiring dialysis, n = 36	12/36 (33)	3/16 (19)	9/20 (45)	0.157
Diabetes, n = 34	16/34 (47)	5/16 (31)	11/18 (61)	0.082
Heart disease, n = 30	9/30 (30)	1/14 (7)	8/16 (50)	0.017
Smoker, n = 27	6/27 (22)	1/16 (6)	5/11 (45)	0.027
Any above underlying conditions or behaviors, n = 38	26/38 (72)	7/15 (47)	19/21 (90)	0.007
Symptoms at onset				
Cough, n = 35	27/35 (77)	14/16 (89)	13/19 (68)	0.244
Fever, n = 38	35/38 (92)	15/17 (88)	20/21 (95)	0.577
Shortness of breath, n = 36	21/36 (58)	8/15 (53)	13/21 (62)	0.607
Any respiratory symptoms at onset, n = 38	35/38 (92)	15/17 (88)	21/21 (100)	0.194
Diarrhea, n = 32	2/32 (6)	1/15 (7)	1/17 (6)	1.000
Clinical course				
Pneumonia, n = 36	30/36 (83)	11/17 (65)	19/19 (100)	0.006
Intubation, n = 32	18/32 (56)	3/15 (20)	15/17 (88)	<0.001
Intensive care, n = 35	23/35 (66)	5/17 (29)	18/18 (100)	<0.001
Age, y	51 (17–84)	39 (17–75)	60 (22–84)	0.001
Onset to hospitalization, d	3 (0–10)	4 (0–10)	2 (0–7)	0.060
Onset to death or discharge, d	17 (1–75)	18 (12–42)	14 (1–75)	0.762

*Values are no./total (%) or median (range). Denominators (total number of patients and total numbers of patients who survived and died) vary by characteristic because information was sometimes unavailable in medical charts.

Twenty-one (55%) of the 38 patients died, all in the hospital. Deceased patients were significantly older than survivors (median age 60.4 vs. 39.4 years; p = 0.001) and were more likely to be men (90% vs. 53%; p = 0.023) and Saudi nationals (76% vs. 35%; p = 0.020). Median time from onset to death or discharge was 17 (range 1.0–84.0) days. HCP patients were more likely than non-HCP patients to be women (54% vs. 12%, p = 0.016), non-Saudi (92% vs. 16%, p<0.001), and younger (median age 37 vs. 65 years; p<0.001); they were also more likely to survive (85% vs. 24%, p<0.001). Two of the 13 HCP patients died. Both were non-Saudi men: a 40-year-old physician with no underlying medical conditions and a 46-year-old information technologist with a history of smoking and hypertension.

Of the 38 MERS-CoV patients reported and investigated during the outbreak period, 33 were associated with 4 facilities (Figure 1). We were unable to link 5 patients epidemiologically to other patients (Table 2).

**Table 2:** Selected characteristics of patients with laboratory-confirmed Middle East respiratory syndrome coronavirus infection, Taif, Saudi Arabia, August 2014–February 2015

Patient no.	Cluster	Healthcare personnel	Date of symptom onset	Date of hospital admission	Date of first positive specimen	Date of death or discharge	Outcome
1	Hospital A	No	2014 Sep 5	2014 Sep 10	2014 Sep 10	2014 Sep 29	Died
2*	Hospital A	No	2014 Sep 13	2008 Jan 1	2014 Sep 13	2014 Sep 20	Died
3	Unlinked	No	2014 Sep 13	2014 Sep 17	2014 Sep 17	2014 Oct 8	Discharged
4	Hospital A	Yes	2014 Sep 15	2014 Sep 21	2014 Sep 21	2014 Oct 27	Discharged
5	Hospital A	No	2014 Sep 17	2014 Sep 24	2014 Oct 5	2014 Oct 17	Died
6	Hospital A	Yes	2014 Sep 23	2014 Sep 23	2014 Sep 23	2014 Oct 3	Died
7	Hospital C	No	2014 Oct 1	2014 Oct 2	2014 Oct 3	2014 Dec 25	Died
8*	Hospital A	No	2014 Oct 3	2014 Jun 30	2014 Oct 6	2014 Dec 15	Died
9	Hospital B	No	2014 Oct 4	2014 Oct 9	2014 Oct 10	2014 Oct 28	Died
10	Hospital C	Yes	2014 Oct 11	2014 Oct 14	2014 Oct 15	2014 Nov 6	Discharged
11†	Hospital B	No	2014 Oct 13	Unknown	2014 Oct 15	2014 Nov 11	Discharged
12	Hospital B	No	2014 Oct 13	2014 Oct 16	2014 Oct 16	2014 Oct 19	Died
13	Hospital B	No	2014 Oct 14	2014 Oct 14	2014 Oct 14	2014 Oct 15	Died
14	Hospital C	Yes	2014 Oct 15	2014 Oct 18	2014 Oct 18	2014 Oct 23	Discharged
15	Hospital B	No	2014 Oct 16	2014 Oct 17	2014 Oct 18	2014 Oct 22	Died
16	Hospital B	No	2014 Oct 16	2014 Oct 23	2014 Oct 23	2014 Oct 30	Discharged
17‡	Hospital B	Yes	–	2014 Oct 25	2014 Oct 25	2014 Oct 30	Discharged
18	Hospital B	No	2014 Oct 16	2014 Oct 18	2014 Oct 27	2014 Oct 27	Died
19	Hospital B	Yes	2014 Oct 17	2014 Oct 27	2014 Oct 27	2014 Nov 2	Discharged
20	Hospital B	Yes	2014 Oct 17	2014 Oct 17	2014 Oct 26	2014 Nov 2	Discharged
21	Hospital C	Yes	2014 Oct 17	2014 Oct 21	2014 Oct 27	2014 Nov 3	Discharged
22	Hospital B	No	2014 Oct 18	2014 Oct 20	2014 Oct 19	2014 Oct 25	Died
23	Hospital B	No	2014 Oct 22	2014 Oct 22	2014 Oct 23	2014 Nov 4	Died
24	Hospital B	Yes	2014 Oct 22	2014 Oct 26	2014 Oct 26	2014 Nov 9	Discharged
25	Hospital B	No	2014 Oct 27	2014 Oct 27	2014 Oct 29	2014 Nov 12	Discharged
26	Hospital B	No	2014 Oct 27	2014 Oct 28	2014 Oct 28	2014 Nov 10	Died
27	Hospital D	No	2014 Nov 3	2014 Nov 1	2014 Nov 3	2014 Nov 10	Died
28‡	Hospital D	Yes	–	2014 Nov 5	2014 Nov 4	2014 Nov 11	Discharged
29	Hospital D	No	2014 Nov 8	2014 Nov 10	2014 Nov 11	2014 Dec 14	Died
30	Hospital D	No	2014 Nov 11	2014 Oct 21	2014 Nov 11	2014 Nov 20	Died
31	Unlinked	No	2014 Nov 15	2014 Nov 19	2014 Nov 20	2014 Dec 8	Died
32	Hospital A	Yes	2014 Nov 20	2014 Nov 20	2014 Nov 22	2014 Nov 27	Died
33	Hospital A	Yes	2014 Nov 24	2014 Nov 27	2014 Nov 27	2014 Dec 11	Discharged
34	Hospital A	No	2014 Nov 27	2014 Dec 2	2014 Dec 4	2014 Dec 25	Discharged
35	Hospital A	Yes	2014 Dec 10	2014 Dec 15	2014 Dec 15	2014 Dec 22	Discharged
36	Unlinked	No	2014 Dec 27	2014 Dec 31	2015 Jan 1	2015 Mar 3	Died
37	Unlinked	No	2014 Dec 28	2015 Jan 7	2015 Jan 6	2015 Jan 19	Discharged
38	Unlinked	No	2015 Jan 4	2015 Jan 9	2015 Jan 11	2015 Jan 20	Died

*Long-term care patient. †Hospitalized only in Riyadh Governorate. ‡Patients had no reported symptoms so no date of onset; they were identified through routine testing of contacts of known patients.

### Hospital A

The first patients in this outbreak were reported from hospital A. Of 10 patients associated with this hospital, 6 had illness onset during September 5–October 2, and 4 had onset during November 20–December 10; patients were tightly clustered in time during these 2 periods (Table 2). The initial patient reported was a 45-year-old male military employee with an unspecified exposure to an outlying farm. Onset of cough, shortness of breath, and fever began on September 5, 2014; he was admitted with respiratory compromise to hospital A on September 10. During September 13–October 2, five additional patients were reported, including 2 HCP employed by the hospital and 2 long-term care inpatients with no community exposures. During November 20–December 10, four additional patients were reported; 3 were HCP. One long-term care patient was admitted to the hospital on June 30, had MERS symptom onset on October 3, and died on December 15, 2014; his hospital course spanned both periods of clustered patients at this facility. Five HCP were among this hospital’s clusters: a 37-year-old male clerk who was married to an intensive-care nurse; a 40-year-old male cleaner; a 29-year-old female nurse; and a 40-year-old male physician and a 46-year-old male working in information technology, both of whom died. Of this hospital’s 10 reported patients, only the 29-year-old nurse had recognized contact with a known MERS-CoV patient before her illness onset. Six (60%) of the 10 patients died during their hospital course: 5 (83%) of 6 patients during the first transmission period and 1 (25%) of 4 patients during the second period.

### Hospital B Dialysis Unit

Hospital B reported 15 patients from its outpatient renal dialysis unit, which was located in a building separate from the acute-care facility. When the outbreak occurred, the dialysis unit had 58 dialysis machines in 8 common rooms, 71 nursing staff, and 377 registered patients receiving periodic hemodialysis. For the 15 patients reported in this cluster, onsets occurred during October 4–27, 2014. Eleven were dialysis patients, and 4 were dialysis unit HCP. The first recognized patient associated with this setting was a 53-year-old man with onset of MERS-related symptoms on October 4, 2015. He underwent dialysis on October 4, 6, 8, and 9 in a 9-bed common room while he was symptomatic. During October 13–28, ten dialysis patients and 3 HCP became ill, and their NP specimens tested positive for MERS-CoV. A fourth HCP reported no symptoms, but his NP specimen was confirmed by rRT-PCR to be MERS-CoV positive on October 25, after RT-PCR screening of NP specimens from identified HCP contacts. Of the 4 MERS-CoV–confirmed HCP, 2 reported working in the dialysis unit while symptomatic on October 18, 20, and 27, just before their MERS-CoV confirmatory testing.

On October 22, infection-control practices were changed on the basis of an onsite assessment by MoH Infection Prevention and Control staff. The changes included screening patients for fever and respiratory symptoms before admission to the dialysis unit; eliminating waiting and prayer areas; discouraging early arrival for dialysis; enforcing a no-visitation policy; increasing distance between patients undergoing dialysis (by reducing number of beds from up to 9 to 6 per room); establishing isolation of dialysis patients with respiratory symptoms; and providing additional infection-control training for staff.

The 15th patient reported from this cluster had illness onset on October 27, after changes were implemented. Of patients in this cluster, 8 (72.7%) of 11 non-HCP died; the 4 HCP survived. Besides the 15 patients reported from this facility, a 17-year-old man who underwent dialysis at this facility on October 4, 6, 8, 11, and 13 reported symptom onset on October 14; his NP specimen was confirmed positive on October 18, after he traveled to Riyadh and was admitted to a hospital there.

### Hospital C

On October 3, a 60-year-old man was transferred to hospital C from an outlying hospital in Taif Governorate after respiratory symptoms developed on October 1 and laboratory testing of his NP specimen confirmed MERS-CoV on October 3. He was transferred to a hospital in Jeddah on October 5 and died there on December 25. On October 11, 15, and 17, three HCP (2 nurses and 1 physician) became ill and were hospitalized at hospital C. Each eventually recovered and was discharged, and no further cases were reported from hospital C.

### Hospital D

On November 1, a 75-year-old woman was transferred to hospital D and admitted to the intensive care unit. She had been evaluated at hospital C on October 22 and November 1 for respiratory complaints and fever. Laboratory testing at hospital D confirmed her NP specimen as MERS-CoV positive on November 3; she was transferred back to Hospital C on November 4 for MERS-CoV treatment and died there on November 9. During HCP contact screening on November 4, an NP specimen from the cleaner of her room at hospital D on November 1–4 was confirmed as positive for MERS-CoV by RT-PCR. He denied symptoms consistent with MERS. On November 8, respiratory symptoms developed in the patient’s 22-year-old grandson; his NP specimen tested positive for MERS-COV on November 11, and he died on December 14. On November 11, an 81-year-old inpatient staying on the same floor where the initial patient received care had onset of respiratory symptoms, and her NP specimen tested positive for MERS-CoV. She died on November 20.

### Additional Cases

Five cases were unlinked to cases reported from the 4 hospitals. The first case-patient was a 65-year-old male retiree with a history of diabetes, heart disease, smoking, and hypertension. After shortness of breath and fever developed on September 13, he sought care at a private hospital on September 17. His NP specimen tested positive for MERS-CoV, and he was referred to hospital C the same day. He was discharged on October 8.

The second case-patient was a 72-year-old male taxi driver with a history of smoking. On November 15, fever developed, followed by sore throat, vomiting, and respiratory failure 2 days later. On November 19, he was admitted to hospital C, where pneumonia was diagnosed, and his NP specimen tested positive for MERS-CoV. He died on December 8.

The third case-patient was a 76-year-old male farmer with a history of diabetes, heart disease, and hypertension. Fever and respiratory symptoms developed on December 27, and he was admitted to hospital C on December 31. MERS-CoV was confirmed by laboratory testing on January 1, and he died on March 3, 2015.

The fourth case-patient was a 33-year-old man who had a history of diabetes and worked as a security guard for a private home. Cough, fever, and headache developed on December 28; on January 7, 2015, he was admitted to hospital C, where pneumonia was diagnosed and his laboratory specimen was MERS-CoV positive. He was discharged on January 19.

The fifth case was a 73-year-old male retiree with diabetes and hypertension. Fever, shortness of breath, nausea, vomiting, and gum bleeding developed on January 4, 2015, and he was admitted to hospital D on January 9 and transferred to hospital C on January 13, 2015. NP specimens collected on January 11 and 19 were positive for MERS-CoV. He died on January 20.

### Laboratory Investigation

#### Molecular Detection and Spike Gene Sequencing

CDC performed laboratory confirmation of MERS-CoV by rRT-PCR on acute-phase serum samples from 17 patients whose NP specimens had been previously confirmed positive for MERS-CoV by RT-PCR (Technical Appendix Table). Median number of days from symptom onset to serum collection was 3.5 (range 0–18 for 16 patients). Serum samples from 15 (88.2%) patients, including 4 samples collected 9–18 days after symptom onset, were confirmed to be positive by rRT-PCR by at least 2 independent assays. The mean rRT-PCR cycle threshold (C_t_) value for a region upstream of the E gene from 9 respiratory specimens was 26.4 (range 17.5–37.9), compared with 34.8 (range 31.1–38.1) for acute serum samples collected on the same day. In general, patients with low C_t_ values (proxies for virus load) in respiratory specimens also had low C_t_ values in serum samples.

**Figure 2:**
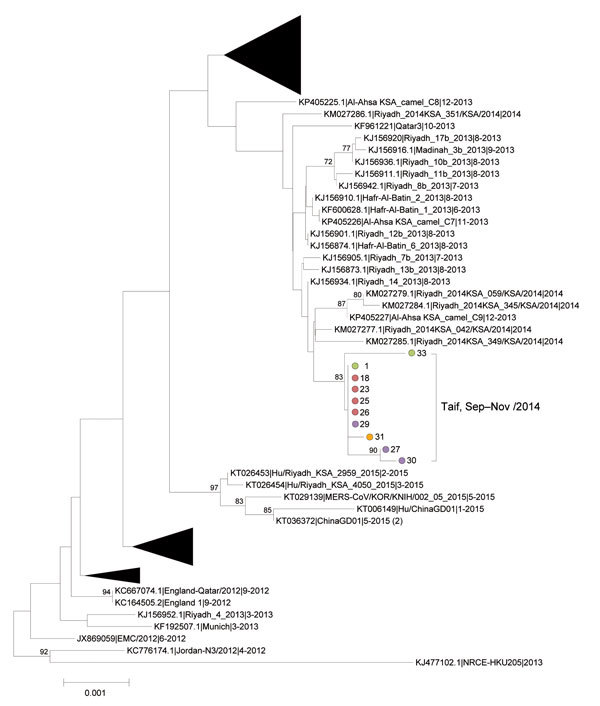
Midpoint-rooted phylogenetic tree inferred from multiple-sequence alignment of 10 new cases of Middle East respiratory syndrome coronavirus (MERS-CoV) spike open reading frame sequences (4,062 nt) from Taif, Saudi Arabia (brackets). Colored circles identify healthcare facilities; numbers indicate individual patients. Taif sequences are shown in context with the closest related sequences that comprise the Hafr-Al-Batin_1 clade, as originally defined by Cotton et al. (*17*), and with sequences related to the 2015 outbreak event in South Korea. For clarity, the remaining published sequences are collapsed into triangles. Published sequences are designated by GenBank accession number, strain name, and month and year of sample collection. The estimated neighbor-joining tree was constructed from nucleotide alignments by using MEGA version 6.06 (http://www.megasoftware.net). Bootstrap support values (1,000 replicates) ≥70% are plotted at the indicated internal branch nodes. Scale bar shows the genetic distance as the number of nucleotide substitutions per site. KSA, Kingdom of Saudi Arabia.

Because of limited available serum volume and generally low virus loads, we focused sequencing efforts on the MERS-CoV spike gene, which has been shown to be a reliable proxy for virus genotyping (*16*) and encodes the receptor-binding domain responsible for attachment to host cells. Sequencing of the spike gene coding region was attempted on all rRT-PCR–positive specimens; complete sequences were obtained from serum samples of 10 patients. The mean N2 rRT-PCR C_t _value of serum samples that were successfully sequenced was 32.8 (range 31.1–35.6), compared with 37.8 (range 35.6–40.6) for samples with failed sequencing. Phylogenetic analysis of the 10 Taif spike sequences showed that the viruses formed a single, discrete cluster located within the Hafr-Al-Batin clade (*17*) and were most closely related to MERS-CoV viruses circulating in Riyadh during 2013 and 2014 (Figure 2). Sequences from 6 patients (1 from hospital A, 4 from hospital B’s dialysis unit, and 1 from hospital D) were identical, and all 10 sequences possessed 2 defining base substitutions at positions 3,670 (G>A) and 3,840 (C>T) (Technical Appendix Table). Sequences from 2 epidemiologically linked cases (patients 27 and 30 in the Technical Appendix Table) associated with hospital D formed a subcluster among the Taif viruses on the basis of 2 defining base substitutions at positions 1,679 (C>T) and 3,496 (G>A).

Five unique nucleotide substitutions conferring predicted amino acid changes were identified among the 10 sequences, of which 3 (E536K, D537E, T560I) were located in the spike protein receptor-binding subdomain that directly interacts with the dipeptidyl peptidase 4 receptor (*18*) (Technical Appendix Table). Random coding changes in the MERS-CoV spike protein may be functionally inconsequential or may confer selective advantage by enabling greater adaptation to the host and possibly enhanced virus transmission (*18*).

#### Serologic Testing

In addition to conducting rRT-PCR on serum samples of 17 patients, CDC performed MERS-CoV serologic assays on these specimens. Specimens from 4 (23.5%) of the 17 patients were considered positive by serologic testing for MERS-CoV antibodies (Technical Appendix Table); 3 of the 4 were positive by ELISA (titers of 6,400), immunofluourescence, and microneutralization (titers of 320). The fourth patient was positive by ELISA (titer of 1,600) and was confirmed positive by microneutralization (titer of 20). Four of 62 HCP exposed to MERS-CoV patients in hospital B’s dialysis unit were positive for antibodies to MERS-CoV, including 1 patient whose specimen was previously confirmed positive by rRT-PCR (Technical Appendix).

## Discussion

Although initial epidemiologic investigation indicated separate transmission events within hospitals A, B, C, and D in Taif Governorate, Saudi Arabia, during September 5–December 15, 2014, sequencing the spike gene coding regions from samples of residual serum from 10 patients indicated a single, discrete cluster. Of the 10 spike sequences from samples collected during September 5–November 9 from patients at 3 facilities, 6 were identical: 1 collected from hospital A, 4 from hospital B’s dialysis unit, and 1 from hospital D. This finding suggests linked transmission among these facilities during this 2-month period. However, the presence of sequences that were not identical to the others may indicate >1 initiating event, even in the same hospital (e.g., patients 1 and 33 in hospital A; Table 2). Despite an exhaustive review of medical charts and interviews with HCP, we could establish no clear epidemiologic links among these facilities, suggesting that unrecognized cases of MERS-CoV infection might not have been captured by the existing surveillance system.

Results from serologic testing of 17 patients showed that 4 were seropositive, despite the relatively short interval between reported onset of illness and collection of serum samples (range 5–7 days). These patients could not be interviewed to confirm exact symptom onset, which may be nonspecific in early MERS-CoV illness. Also, 1 of the 4 seropositive specimens could not be confirmed by rRT-PCR at CDC but was found to be rRT-PCR positive in Saudi Arabia.

These findings highlight the challenges and limitations of epidemiologic investigations of MERS and show the value of molecular techniques. In addition to standardized data collection, viral sequencing should be attempted when possible to enable better understanding of transmission events. Our investigation shows the highly infectious nature of MERS-CoV, including high rates of illness and death from within dialysis settings, as previously noted (*8**,**9*). In hospital B’s dialysis unit, 15 persons had MERS-CoV infections confirmed by rRT-PCR during a 3-week period. Eleven of the 15 were non-HCP patients who were regularly undergoing dialysis, and 8 (73%) died. The other 4 were HCP, 3 of whom were symptomatic. Our subsequent serologic investigation of HCP in the dialysis unit identified 3 additional and previously unrecognized HCP who were seropositive but denied symptoms at interview (Technical Appendix). A total of 18 persons were involved in the dialysis unit transmission event. Although this investigation did not firmly establish modes of transmission, risk for respiratory droplet transmission in this setting might have been increased because of close spacing (<2 meters between beds) of patients who also were likely to be immunocompromised by end-stage renal disease and other underlying conditions such as diabetes. After implementation of recommended changes in infection-control practices, the number of cases reported in association with this dialysis unit quickly declined.

Our investigation is subject to several limitations. Our team had limited access to hospital A, although we were able to assess case reporting and investigation forms, discuss patients with providers, and receive patient specimens for further laboratory testing. This outbreak occurred among at least 4 facilities, and contact investigations were conducted by those facilities. Although the contact investigations were critical for detecting mildly ill patients in this outbreak, contact tracing and testing might not have been uniformly conducted in all facilities, potentially limiting the scope of our investigation. Although we were able to obtain partial MERS-CoV genome sequences from acute-phase serum samples from 10 of 12 patients, specimens were not available for all patients. Additional viral sequences from the unlinked cases would have been particularly useful in understanding whether these cases were possibly linked to the identified facility transmission events. The limited availability of specimens restricted our ability to obtain full-genome sequences that would likely provide greater epidemiologic power in resolving transmission events. Although we attempted to link the results of our epidemiologic investigation with the spike gene sequences from investigated cases, we cannot be certain whether the virus was introduced into the healthcare environment in Taif on one or multiple occasions. Circulation of MERS-CoV among camels in Taif has been documented (*19*), and the detection of phylogenetically common or closely related viruses in the human cases in this investigation might reflect multiple introductions of the same or similar viruses circulating in camels in Taif during this outbreak period. Notably, the 6 patients with identical spike gene sequences were in 3 clusters and had onset dates that spanned 64 days. 

A comparison of the sensitivities of the MERS nucleocapsid ELISA and the MERS spike ELISA has not been published. Additional evaluation to better characterize the clinical sensitivity and specificity of the MERS-CoV serologic assays used in this study is necessary, and systematic cross-validation will be needed in the future.

Repeated introduction of MERS-CoV into healthcare facilities, resulting in transmission among patients, visitors, and HCP, has been a defining feature of MERS-CoV epidemiology since its emergence in 2012. Our investigation shows the persistence of MERS-CoV circulation in multiple healthcare settings over an extended period, despite lack of clearly defined epidemiologic links, and underscores the importance of identifying and monitoring exposed HCP, patients, and visitors. MERS-CoV transmission in any healthcare facility should trigger increased vigilance among all healthcare facilities that could potentially share patients and staff. Increased understanding of epidemiologic links among identified patients during transmission events is needed to inform surveillance strategies and infection prevention and control.

**Technical Appendix.** Details of a healthcare worker serosurvey in the dialysis unit of hospital B and laboratory results of patients with confirmed MERS-CoV, Taif, Saudi Arabia, August 2014–February 2015.
