# Supplementary material for: Multifacility Outbreak of Middle East Respiratory Syndrome in Taif, Saudi Arabia
Source: Emerg Infect Dis. 2016 Jan;22(1):32–40. doi: 10.3201/eid2201.151370 (PMC4696715; doi:10.3201/eid2201.151370)
Supplement: Supplementary file 1 — Technical Appendix. Details of a healthcare worker serosurvey in the dialysis unit of hospital B and laboratory results of patients with confirmed MERS-CoV, Taif, Saudi Arabia, August 2014–February 2015. [file 15-1370-Techapp-s1.pdf]

Article DOI: http://dx.doi.org/10.3201/eid2201.151370

Multifacility Outbreak of Middle East Respiratory Syndrome in Taif, Saudi Arabia

Technical Appendix

Details of Healthcare Worker Serosurvey in the Dialysis Unit of Hospital B

In October 2014, fifteen cases of MERS-CoV infection were identified among outpatients (n = 11) and staff (n = 4) at the dialysis unit of Hospital B in Taif, Saudi Arabia. Hospital B is a 500-bed tertiary Ministry of Health hospital in Taif Governorate, Makkah Region, Saudi Arabia, and has an associated but physically separate outpatient renal dialysis unit. Serologic analysis was performed on serum samples of PCR-positive MERS patients and of healthcare personnel (HCP) in the dialysis unit of Hospital B. HCP with potential exposure to MERS patients were identified. Enrolled HCP provided written informed consent and completed a brief, standardized questionnaire to gather information about demographics, job duties, symptom information, and specific exposures to MERS-CoV patients during the period of suspected transmission. Following a period of >3 weeks, a blood specimen (<20 mL) was collected from each HCP for serologic detection of antibodies to MERS-CoV. Blood specimens were centrifuged to separate sera, which were transported to the Saudi Arabia Ministry of Health and to CDC for antibody and rRT-PCR testing. Demographic and clinical characteristics were reported, and differences were assessed for significance ($p = 0.05$) by using χ^2 test, Fisher exact test, and *t*-test, as appropriate. All data were analyzed by using SAS version 9.3 (SAS Institute, Cary, NC, USA).

Seventy HCP were identified as having had exposure to the dialysis unit of Hospital B during the period of suspected transmission (October 1–31). Interviews by using a standardized questionnaire were conducted and serum samples collected on November 24, 2014. Of the 70 identified HCP, 62 (89%) provided serum specimens, and 38 (54%) completed exposure assessments. Demographics of respondents with completed assessments and those without assessments did not were similar. For the 38 respondents with available information, the median age was 32 years (range 25–61). Twenty-five (66%) of the 38 were female, and 29 (76%) were

nurses. The 38 respondents also included 5 (13%) housekeeping staff, 2 (6%) biomedical technicians, 1 (3%) physician, and 1 (3%) pharmacist. Most (82% and 79%, respectively) came within 1 meter of a patient, touched a patient, or both.

Serum samples from 4 (6.5%) of 62 exposed HCP (2 nurses, 1 physician, and 1 housekeeper) were positive by the MERS-CoV N ELISA used for initial MERS-CoV screening, and all were confirmed positive by immunofluourescence and microneutralization assays. Of the 4 identified seropositive HCP, 3 denied any assessed symptom and had not been previously recognized as infected with MERS-CoV. The remaining seropositive HCP was 1 of four previously identified symptomatic HCP from the dialysis unit of Hospital B. Of the remaining 3 previously identified rRT-PCR–positive, dialysis-related HCP, 2 completed exposure assessments but declined blood draw, and 1 was unavailable for exposure assessment or serologic testing. Of 62 serum samples from the dialysis unit HCP serosurvey, all were rRT-PCR negative.

Of the 70 HCP identified as having exposure to the dialysis unit during the transmission period, 7 (10%) had rRT-PCR or serologic evidence of MERS-CoV infection; 5 of these 7 completed exposure assessments. Among the 38 HCP who completed exposure assessments and provided serum specimens, demographic information and exposures were similar between seropositive and seronegative participants (data not shown).

Our serologic investigation of the dialysis unit HCP identified 3 additional, previously unrecognized seropositive HCP, who denied symptoms at interview. Overall, 18 persons (11 patients and 7 HCP) were found to be involved in the dialysis unit transmission event. Our serosurvey of HCP in the dialysis unit was limited by the small number of seropositive participants and did not reveal risk factors for transmission among HCP.

References

1. Wang N, Shi X, Jiang L, Zhang S, Wang D, Tong P, et al. Structure of MERS-CoV spike receptor-binding domain complexed with human receptor DPP4. Cell Res. 2013;23:986–93. PubMed http://dx.doi.org/10.1038/cr.2013.92

Technical Appendix Table. Laboratory results obtained from patients with confirmed Middle East respiratory syndrome coronavirus, Taif, Saudi Arabia, August 2014–February 2015*

Patient no.	Specimen collection date	rRT-PCR result: Respiratory sample (upE/ORF1a C_t)†	rRT-PCR result: Serum sample (upE/N2/N3 C_t)‡	Spike sequence§	Location of spike gene open reading frame nucleotide and amino acid substitution¶: 795	1,606 (536)	1,611 (537)	1,679 (560)	2,142	3,496 (1,166)	3,543	3,670 (1,224)	3,840	GenBank accession no.	Serology#
1	2014 Sep 10	NA/17.8	33.8/32.8/NA	Y	–	–	–	–	–	–	–	G>A (G>S)	C>T	KR912191	Pos
5	2014 Oct 5	26.9/26.5	36.3/35.6/NA	N	NA	NA	NA	NA	NA	NA	NA	NA	NA	NA	Neg
18	2014 Oct 27	25.6/25.7	32.6/32.5/NA	Y	–	–	–	–	–	–	–	G>A (G>S)	C>T	KR912190	Neg
22	2014 Oct 19	33.9/34.3	37.3/37.8/NA	N	NA	NA	NA	NA	NA	NA	NA	NA	NA	NA	Neg
23	2014 Oct 24	NA	31.7/31.4/NA	Y	–	–	–	–	–	–	–	G>A (G>S)	C>T	KR912192	Neg
24	2014 Oct 27	29.1/29.3	Neg/Neg/NA	N	NA	NA	NA	NA	NA	NA	NA	NA	NA	NA	Pos
25	2014 Oct 27	NA	33.1/33.3/NA	Y	–	–	–	–	–	–	–	G>A (G>S)	C>T	KR912193	Neg
26	2014 Oct 27	NA	32.9/32.2/NA	Y	–	–	–	–	–	–	–	G>A (G>S)	C>T	KR912194	Neg
27	2014 Nov 4	20.7/21.3	31.1/31.2/NA	Y	–	–	–	C>T (T>I)**	–	G>A (A>T)	–	G>A (G>S)	C>T	KR912188	Neg
28	2014 Nov 6	34.7/31.4	Neg/Neg/NA	N	NA	NA	NA	NA	NA	NA	NA	NA	NA	NA	Neg
29	2014 Nov 11	21.6/21.7	31.7/31.1/NA	Y	–	–	–	–	–	–	–	G>A (G>S)	C>T	KR912189	Neg
30	2014 Nov 12	23.7/24.5	35.6/35.6/NA	Y	–	–	–	C>T (T>I)**	C>T	G>A (A>T)	–	G>A (G>S)	C>T	KR912195	Neg
31	2014 Nov 22	30.0/30.5	36.5/32.2/NA	Y	–	G>A (E>K)**	–	–	–	–	–	G>A (G>S)	C>T	KR912196	Pos
33	2014 Nov 28	Neg/Neg	36.1/35.6/NA	Y	C>T	–	C>A (D>E)**	–	–	–	T>C	G>A (G>S)	C>T	KR912187	Neg
36	2015 Jan 2	23.0/23.5	Neg/37.1/35.5	N	NA	NA	NA	NA	NA	NA	NA	NA	NA	NA	Pos
37	2015 Jan 9	37.9/37.9	38.1/37.8/NA	N	NA	NA	NA	NA	NA	NA	NA	NA	NA	NA	Neg
38	2015 Jan 13	36.9/35.8	Neg/40.6/38.8	N	NA	NA	NA	NA	NA	NA	NA	NA	NA	NA	Neg

*C_t, cycle threshold values; MERS-CoV, Middle East respiratory syndrome coronavirus; NA, not available; Neg, negative; ORF, open reading frame; Pos, positive; rRT, real-time reverse transcription; upE, upstream of E gene; –, no nucleotide substitution.
†rRT-PCR assays targeting MERS-CoV upE and ORF1a (replicase) performed on patient respiratory samples at the Ministry of Health Regional Laboratory at Makkah, Saudi Arabia.
‡rRT-PCR assays targeting MERS-CoV upE and N2/N3 (nucleocapsid genes) performed on patient acute phase serum samples at the Centers for Disease Control and Prevention, Atlanta, Ga, USA.
§From serum sample. Y, sequenced; N, not sequenced.
¶Nucleotide and predicted amino acid (in parentheses) substitutions are unique to the Taif spike open reading frames as compared with 146 published spike sequences. The spike open reading frame sequence obtained from patient 33 also contained a single synonymous nucleotide substitution at position 1,335 (T>C) that differed from other Taif sequences but that was present among some published sequences (not shown in this Table). Spike open reading frame nucleotides 1–4,062 and amino acids, 1–1,353.
#MERS-CoV antibody positivity was defined as a positive result by the MERS-CoV N ELISA and confirmatory positive results by the MERS-CoV immunofluourescence and microneutralization assays. Serum samples that were negative by MERS-CoV N ELISA were considered negative for MERS-CoV antibodies.
**Glu536Lys, Asp537Glu and Thr560Ile located in the spike protein receptor-binding subdomain as described by Wang et al. (*1*).
